# *Plasmodium yoelii* infection inhibits murine leukaemia WEHI-3 cell proliferation in vivo by promoting immune responses

**DOI:** 10.1186/s40249-018-0433-4

**Published:** 2018-05-16

**Authors:** Zhen-Zhen Tong, Zheng-Ming Fang, Qi Zhang, Yun Zhan, Yue Zhang, Wan-Fang Jiang, Xiao Hou, Yong-Long Li, Ting Wang

**Affiliations:** 1 Department of Parasitology, School of Basic Medicine, Tongji Medical College, Huazhong University of Science and Technology, Wuhan, Hubei China; 2 Department of Immunology, School of Basic Medicine, Tongji Medical College, Huazhong University of Science and Technology, Wuhan, Hubei China

**Keywords:** Leukaemia, *Plasmodium yoelii*, WEHI-3 cells, Anti-leukaemia

## Abstract

**Background:**

Leukaemia is a malignant leukocyte disorder with a high fatality rate, and current treatments for this disease are unsatisfactory. Therefore, new therapeutic strategies for leukaemia must be developed. Malaria parasite infection has been shown to be effective at combating certain neoplasms in animal experiments. This study is to demonstrate the anti-leukaemia activity of malaria parasite *Plasmodium yoelii* (*P. yoelii*) infection,.

**Methods:**

In this study, the proportion of CD3, CD19, CD11b and Mac-3 cells was analysed by flow cytometry; the levels of IFN-γ and TNF-α in individual serum samples were measured by enzyme-linked immunosorbent assay, and the phagocytic activity of macrophages and natural killer (NK) cell activity were measured by flow cytometry.

**Results:**

We found that *P. yoelii* infection significantly attenuated the growth of WEHI-3 cells in mice. In addition, tumor cell infiltration into the murine liver and spleen was markedly reduced. We also demonstrated that malaria parasite infection elicited anti-leukaemia activity by promoting immune responses, including increasing the surface markers of T cells (CD3) and B cells (CD19); decreasing the surface markers of monocytes (CD11b) and macrophages (Mac-3); inducing the secretion of IFN-γ and TNF-α; and increasing NK cell and macrophage activity.

**Conclusions:**

Malaria parasite infection significantly decreases the number of myeloblasts and inhibits neoplasm proliferation in mice. In addition, malaria parasite infection inhibits murine leukaemia by promoting immune responses.

**Electronic supplementary material:**

The online version of this article (10.1186/s40249-018-0433-4) contains supplementary material, which is available to authorized users.

## Multilingual abstracts

Please see Additional file 1 for translations of the abstract into the five official working languages of the United Nations.

## Background

Leukemia comprises a group of malignant hematologic disorders. The disease incidence is approximately 15/100 000 individuals. In the United States and China, leukaemia accounts for the greatest proportion of childhood cancer cases [1, 2]. Current treatments for the disease remain unsatisfactory; therefore, new therapies must be established.

Recently, several researchers have reported an adverse relationship between parasitic infections and cancer. Parasitic infections, such as *Trypanosoma cruzi* [3], *Toxoplasma gondii* [4], *Trichinella spiralis* [5, 6], *Toxocara canis* [7], *Acanthamoeba castellanii* [8] and *Plasmodium yoelii* infections [9], have reportedly inhibited cancer growth in animal experiments. In addition, the adverse relationship between parasitic infections and cancer in humans has been demonstrated in epidemiological investigations of the prevalence of parasitic infections and cancer diseases [10]. In 1980, statistical data from the WHO indicated that the incidence of cancer was lowest in malaria-endemic areas [11].

Although the mechanisms underlying the anticancer activity of some parasites are unclear, the mechanisms may be associated with antigens that are shared by parasites and cancer; these antigens may increase immune responses, which may nonspecifically induce anticancer activities [10]. In the present study, we examined the anti-leukaemia activity of *P. yoelii* infection in mice bearing WEHI-3 leukaemia cells and found that malaria parasite infection significantly attenuated WEHI-3 cell proliferation in these mice. We also demonstrated that *P. yoelii* infection induced anti-leukaemia activity by promoting immune responses.

## Methods

### Mice and parasites

We obtained 8- to 10-week-old female BALB/c mice from the Experimental Animal Center at Tongji Medical College (China; rodent license no. SYXK (e) 2010–0057). The animals were housed under specific-pathogen-free conditions in the animal facility and provided a sterile diet and autoclaved water. The animals were acclimated for 1 week prior to starting the experiment. All experimental procedures involving animals were approved by the Animal Research Ethics Committee of Tongji Medical College and performed in accordance with the institutional guidelines for the humane and ethical care of animals.

The *P. yoelii* 17XNL strain was obtained from Third Military Medical University in China.

### Murine WEHI-3 leukaemia cells

The murine WEHI-3 myelomonocytic leukaemia cell line was obtained from the Cell Resource Center of the Institute of Basic Medical Sciences at the Chinese Academy of Medical Sciences. Cells were cultured in high-glucose DMEM containing 10% FBS, 100 units/ml penicillin, 100 μg/ml streptomycin and 2 mmol/L L-glutamine at 5% CO_2_ and 37 °C.

### Establishment of the murine leukaemia model and infection with the malaria parasite

A murine leukaemia model was established as described by He and Na [12]. A total of 40 BALB/c mice were divided into four groups (10 animals per group). Group I (con) consisted of control mice; group II (Py*)* mice were intraperitoneally (i.p.) inoculated with 1 × 10^5^*P. yoelii* 17XNL-parasitized erythrocytes; group III (WEHI-3) mice were i.p. inoculated with 1 × 10^5^ WEHI-3 cells; and group IV (WEHI-3 + Py) mice were i.p. inoculated with 1 × 10^5^ WEHI-3 cells and then i.p. inoculated with 1 × 10^5^*P. yoelii* 17XNL-parasitized erythrocytes 1 week later.

All mice were euthanized under anesthesia 2 weeks after inoculation with *P. yoelii* 17XNL. Each mouse was anesthetized by i.p. administration of 0.67% pentobarbital sodium at a dose of 100 μl/10 g body weight. No spontaneous deaths occurred before the mice were sacrificed. The blood, livers and spleens were collected from the mice, and bone marrow was flushed from the femurs of the sacrificed mice.

### Bone marrow smear and histopathological examination

All bone marrow was flushed from the femurs of the sacrificed mice and smeared as described by Alabsi et al. [13]. Leukocyte classification based on cell morphology was performed by Wright’s staining of the bone marrow smears, and the myeloblast percentages were determined by counting 500 nucleated bone marrow cells under a microscope.

Isolated spleen and liver samples were fixed in 4% formaldehyde, embedded in paraffin and sectioned at a thickness of 5 μm. The sections were stained with hematoxylin and eosin (H&E) in accordance with the procedures described by Chung et al. [14] and were used for histopathological examination.

### Assay of natural killer (NK) cell activity

Splenocytes were isolated from the fresh spleens of each mouse in all groups, and approximately 1 × 10^7^ splenocytes were cultured in each well of 24-well culture plates. YAC-1 cells (NK target cells) obtained from the Laboratory of Cell Engineering of Tongji Medical College were stained according to the manufacturer’s protocol (PKH67 Fluorescent Cell Linker Kits, Sigma-Aldrich Corp). Approximately 1 × 10^7^ splenocytes from each mouse were mixed with labeled YAC-1 cells in the wells of a 96-well plate in an atmosphere containing 5% CO_2_ at 37 °C; the effector/target cell ratio was 50:1 or 100:1. After 12 h of incubation, NK cell activity was determined using a PI exclusion assay and flow cytometry (BD LSR II, USA) as described by Lin et al. [15].

### Assay of macrophage phagocytosis

Macrophages were isolated from the peritoneal cavities of the control and experimental animals to investigate phagocytosis using a PHAGOTEST kit (Invitrogen V-6694 Vybrant®, USA). Subsequently, for each mouse, a 0.5 ml cell suspension (with 2 × 10^6^ isolated cells/mL) in RPMI 1640 medium with 10% FBS was individually incubated with opsonized fluorescein isothiocyanate (FITC)-labeled *E. coli* (0.5 ml) for 1.5 h at 37 °C. In accordance with the manufacturer’s instructions, ice-cold quenching solution (100 μl) was added to stop the reaction. After completion of phagocytosis, the macrophages were analysed using a flow cytometer (BD LSR II, USA). Fluorescence data were collected for 10 000 cells and analysed using Cellquest software.

### Assay of leukocyte surface markers

For leukocyte collection, a blood sample from each mouse was immediately treated with ammonium chloride to lyse the red blood cells. The sample was then centrifuged at 1500 rpm at 4 °C for 15 min. After centrifugation, the supernatant was discarded, and the white blood cells were harvested. To measure cell surface markers of T cells (CD3), B cells (CD19), monocytes (CD11b) and macrophages (Mac-3), isolated white blood cells were stained with an anti-CD3-FITC antibody, an anti-CD19-phycoerythrin (PE) antibody, an anti-CD11b-FITC antibody and an anti-Mac-3-PE antibody (eBioscience, USA). The cell markers were then analyzed by flow cytometry (BD LSR II, USA) as described by Lin et al. [15].

### Cytokine assay

The levels of the Th1-type cytokines IFN-γ and TNF-α were measured in each serum sample according to the instructions of the ELISA kit manufacturer (eBioscience, USA). The OD values of the reactions were read at 450 nm in an ELISA reader. The cytokine levels in the samples were calculated using standard curves constructed with known amounts of mouse recombinant IFN-γ and TNF-α (eBioscience, USA), and the results are expressed in picograms per ml.

### Statistical analysis

The data are expressed as the mean ± *SD*, and differences between the control and experimental groups were analysed by one-way ANOVA. A value of *P* < 0.05 was considered statistically significant.

## Results

### Bone marrow smear and histopathological examination

**Fig. 1:**
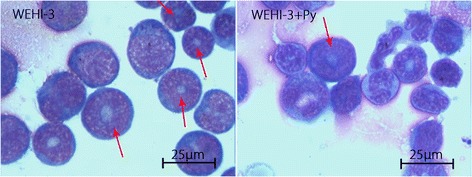
Myeloblasts in bone marrow smears from mice in the WEHI-3 and WEHI-3 + *P. yoelli* (Py) groups. Mice were i.p. injected with WEHI-3 cells. After 3 weeks, the bone marrow was flushed from the femurs of the sacrificed mice and smeared. Leukocyte classification based on cell morphology was performed by Wright’s staining of the bone marrow smears. The arrows (↓) indicate neoplasm cells (myeloblast), which contain large, irregular nuclei accompanied by prominent nucleoli and abundant light eosinophilic cytoplasm

**Fig. 2:**
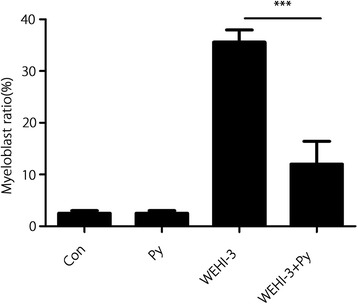
Percentages of myeloblasts in bone marrow smears from mice of each group. The percentages of myeloblasts were determined by evaluating 500 nucleated bone marrow cells. The experiments were performed twice in duplicate, producing similar results; *** *P* < 0.001

Analysis of bone marrow smears showed that myeloblasts were present in the bone marrow of all mice (Fig. 1), but the percentage of myeloblasts in the mice inoculated with WEHI-3 cells was much higher than that in the mice of other groups (*P* < 0.001) (Fig. 2). After incubation with *P. yoelii* 17XNL, the percentage of myeloblasts in the mice inoculated with WEHI-3 cells decreased significantly (Fig. 2).

**Fig. 3:**
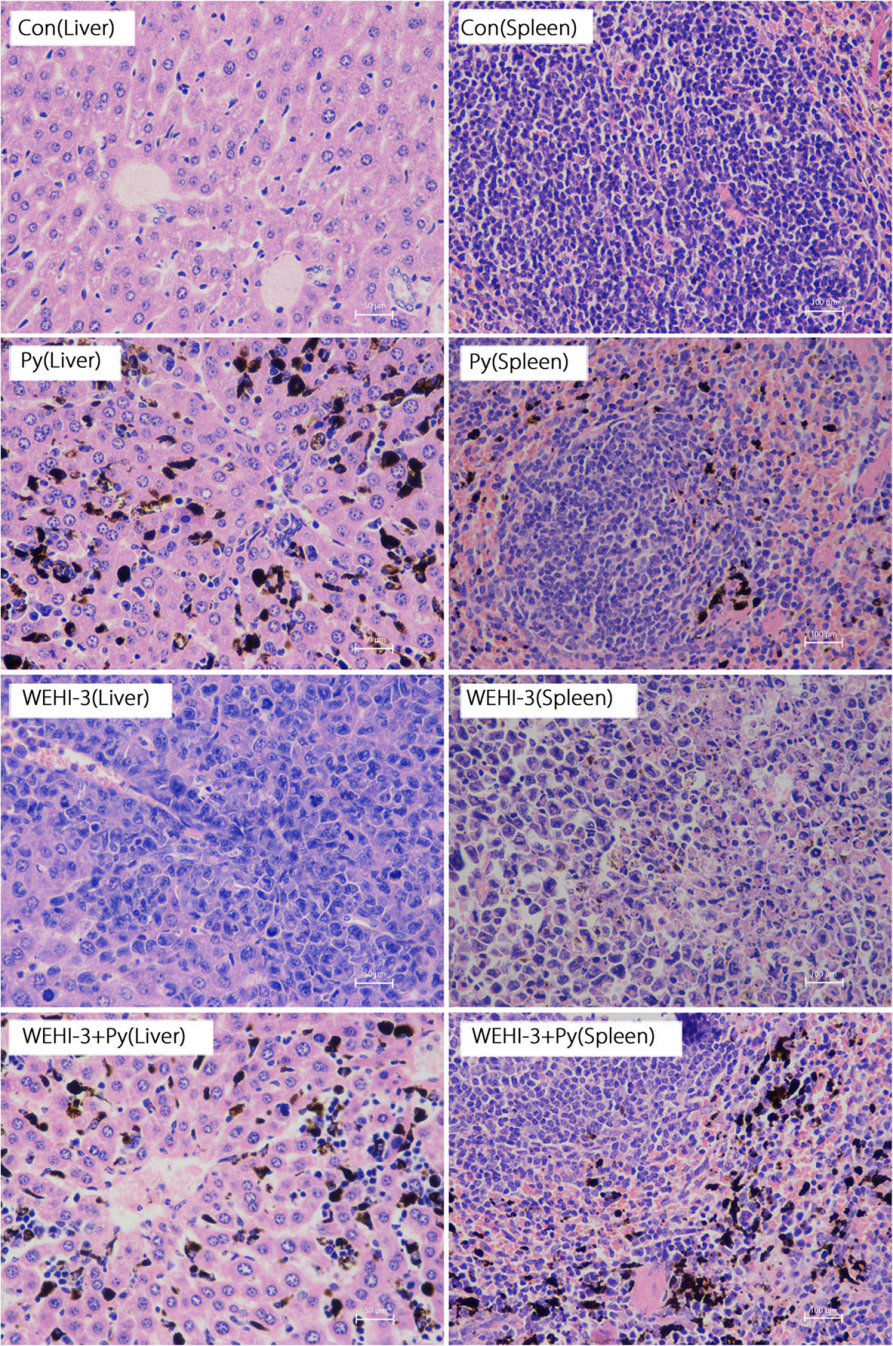
Histopathological examination of liver and spleen tissues from each group. The isolated spleen and liver samples were fixed in 4% formaldehyde, embedded in paraffin and sectioned. The sections (5 μm) were stained with H&E and evaluated under a microscope at 400 × magnification. The Con group shows normal structures and no infiltrated neoplasm cells in the liver and spleen. The *P. yoelli* (Py) group shows normal structures and no infiltrated neoplasm cells but shows deposition of malaria pigments in the liver and spleen. The WEHI-3 group shows abundant infiltrated neoplasm cells in the liver and spleen. The WEHI-3 + Py group shows markedly reduced neoplasm cell infiltration in the liver and spleen

**Fig. 4:**
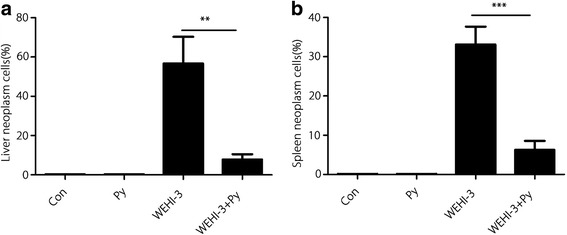
Degree of neoplasm cell infiltration in the livers (**a**) and spleens (**b**) of mice. The mean proportion of neoplasm cells was calculated from five randomly selected fields by scanning the tissue sections at high power using an Olympus CX31 microscope (400 × magnification). The results are expressed as the mean ± *SD*, and samples were obtained from 10 mice per group. ** *P* < 0.01 and *** *P* < 0.001

The histopathology results for liver and spleen tissues are shown in Figs. 3 and 4. In the WEHI-3 group, abundant neoplastic cells were found in liver and spleen tissues (Fig. 3), whereas the number of these cells were markedly reduced in the WEHI-3 + Py group (*P* < 0.01 and *P* < 0.001 compared with the liver (Fig. 4a) and spleen (Fig. 4b) tissues of the WEHI-3 group, respectively). The results indicated that malaria parasite infection could inhibit the development of leukaemia in mice.

### Effect of *P. yoelii* infection on NK cell activity

**Fig. 5:**
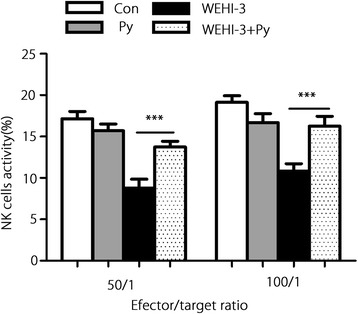
Effect of *P. yoelii* infection on NK cell activity. Splenocytes were isolated from all mice in all groups. NK cell cytotoxicity was determined at the indicated effector-to-target ratios using a PI exclusion assay and flow cytometry. The results are expressed as the mean ± *SD* of 10 samples per group; *** *P* < 0.001

The activity of NK cells isolated from the mouse spleens was measured, and the results showed that NK cell activity against YAC-1 target cells was significantly increased at target ratios of 50:1 and 100:1 in the WEHI-3 + Py group compared with the WEHI-3 group (Fig. 5), indicating that malaria parasite infection could increase NK cell cytotoxicity in WEHI-3 cell-bearing mice.

### Effect of *P. yoelii* infection on the phagocytic activity of macrophages

**Fig. 6:**
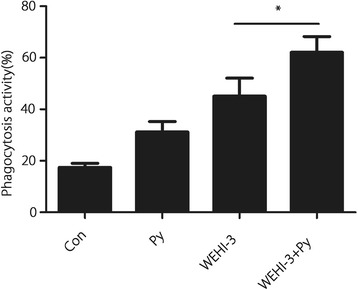
Effect of *P. yoelii* infection on the phagocytic activity of macrophages. Macrophages were isolated from the peritoneal cavities of the mice, and the phagocytic activity of these macrophages was analysed using a flow cytometer. The results are expressed as the mean ± *SD* of 10 samples per group; * *P* < 0.05

Phagocytosis by macrophages isolated from the peritoneal cavity of mice in each group was evaluated using a flow cytometer, and the results shown in Fig. 6 indicate that malaria parasite infection promoted the phagocytic activity of macrophages in WEHI-3 cell-bearing mice.

### Surface markers of leukocytes in mice

**Fig. 7:**
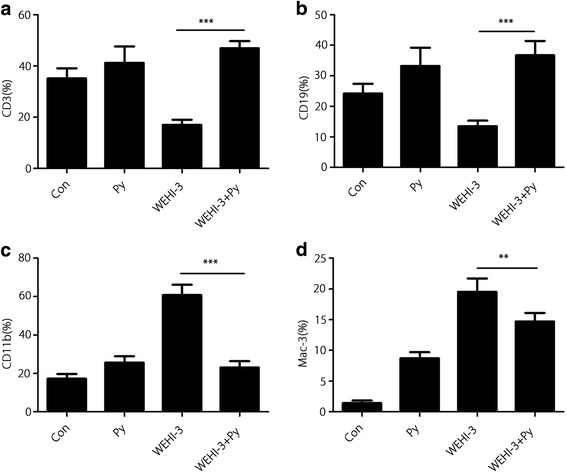
Surface markers of leukocytes. White blood cells were collected from individual mice, and the specific cell surface markers CD3 (**a**), CD19 (**b**), CD11b (**c**) and Mac-3 (**d**) were analysed by flow cytometry. The results are expressed as the mean ± *SD*, and samples were obtained from 10 mice per group. ** *P* < 0.01 and *** *P* < 0.001

CD3, CD19, CD11b and Mac-3, the surface markers of leukocytes, were analysed in each group using flow cytometry. The data presented in Fig. 7 show that *P. yoelii* infection significantly increased the levels of CD3 (Fig. 7a) and CD19 (Fig. 7b) but decreased the levels of CD11b (Fig. 7c) and Mac-3 (Fig. 7d) compared with those levels in the WEHI-3 group. These results indicated that malaria parasite infection promoted differentiation of T and B cell precursors and inhibited differentiation of monocyte and macrophage precursors.

### Cytokine assays

**Fig. 8:**
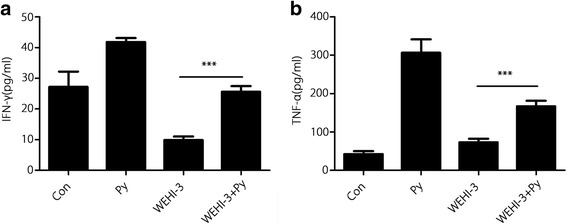
*P. yoelii* infection induced the production of IFN-γ and TNF-α. The levels of IFN-γ (**a**) and TNF-α (**b**) in sera were measured by ELISA. The results are expressed as the mean ± *SD*, and samples were obtained from 10 mice per group; *** *P* < 0.001

Changes in cytokine production in the sera from all groups are shown in Fig. 8a and b. *P. yoelii* infection (WEHI-3 + Py group) significantly increased the levels of the Th1-type cytokines IFN-γ and TNF-α compared with those in the WEHI-3 group (*P* < 0.001).

## Discussion

Leukaemia is an aggressive myeloid neoplasm characterized by arrest of myelopoietic cell maturation leading to myeloblast accumulation in the bone marrow and/or blood.

Leukaemia is diagnosed when myeloblasts constitute at least 20% of the nucleated cells in bone marrow or blood [16]. Murine monomyelocytic leukaemia cells (WEHI-3) were originally derived from BALB/c mice [17]. The major characteristics of the WEHI-3 leukaemia mice were elevated levels of peripheral monocytes and granulocytes with immature morphology, clearly enlarged and infiltrated spleens, and large numbers of undifferentiated cells in the bone marrow [12]. Therefore, the animal model fulfilled all the criteria of human myelomonocytic leukaemia [17].

In the present study, we demonstrated that myeloblasts accounted for > 30% of the non-erythroid cells in the bone marrow of mice inoculated with WEHI-3 cells, and we found neoplastic cells in the spleens and livers of these mice through histopathological examination. These changes indicated that the leukaemia mouse model was successfully established. After the WEHI-3 cell-bearing mice were inoculated with *P. yoelii*, the percentage of myeloblasts in the bone marrow was less than 20%, and the number of neoplastic cells were notably decreased in the spleens and livers (*P* < 0.001 and *P* < 0.01, respectively). These results suggested that malaria parasite infection significantly decreased the number of myeloblasts and inhibited neoplasm proliferation in these mice.

Malaria is a parasitic disease caused by organisms in the *Plasmodium* genus, and an adverse relationship between parasitic infections and cancer has been reported [10]. Additionally, researchers have demonstrated that the human malaria parasite induces periodic high fever in the acute phase and that high fever can inhibit tumour growth [18]. However, the *P. yoelii* 17XNL strain does not induce fever in rodent malaria [19], and we did not observe fever in the animals in our study (data not shown). Therefore, the anti-leukaemia effect of *P. yoelii* 17XNL infection is not associated with hyperthermia.

The mechanisms of tumour resistance induced by parasites are not well understood, but various mechanisms may be involved, such as the presence of antigens common to tumours and parasites or concomitant immunity induced by living parasites such that tumours may be mistaken as parasites and subjected to an immune response [17]. In preliminary experiments, we used *P. yoelii* parasite lysates, but no anti-leukaemia activity was observed in the mice (data not shown), suggesting that the quantity of common antigens is very low in crude lysates of *P. yoelii* and that the virus must be extracted and purified. Additionally, this finding may also imply that the anti-leukaemia activity of *P. yoelii* infection is induced by living parasites. In fact, protective immunity to malaria is primarily a form of concomitant immunity and is effective only against residual populations of parasites [20]; therefore, lysates of *P. yoelii* are ineffective.

Malaria parasite infection can stimulate the host immune system, inducing polyclonal activation and massive proliferation and differentiation of lymphocytes with parasite-unrelated specificities [21, 22]. In the present study, we demonstrated that *P. yoelii* infection could significantly decrease the percentages of CD11b and Mac-3 cells in the blood of mice inoculated with WEHI-3 cells, indicating that the differentiation of macrophage and monocyte precursors was inhibited. Meanwhile, *P. yoelii* infection increased the percentages of CD3 and CD19 cells in the mice, indicating that differentiation of the T and B cell precursors was enhanced. These results are consistent with those from other reports [23, 24]. Stimulation of T and B cell proliferation is believed to be efficacious in combating some cancers, including myeloid leukaemia [25–27]. We also found that malaria parasite infection could induce IFN-γ and TNF-α secretion, which may lead to improvements in the treatment of some cancers by inducing a massive influx of inflammatory cells as well as the production of Th1 cytokines [28–30]. *P. yoelii* infection enhanced T and B cell proliferation and increased the levels of the Th1 IFN-γ and TNF-α, suggesting that adaptive immunity is involved in the anti-leukaemia effect. Meanwhile, *P. yoelii* infection promoted NK cell activity and macrophage phagocytosis; these scavenger cells are widely believed to be an essential component of host immune defences against myeloid leukaemia [25, 31, 32], indicating that innate immunity is also involved in the anti-leukaemia effects from *P. yoelii* infection.

In the present study, we found that the percentage of macrophages was decreased (Fig. 7) but the activity of macrophages was increased (Fig. 6) in the *P. yoelii*-infected mice bearing WEHI-3 cells compared to the non-infected mice bearing WEHI-3 cells. These results seem to be contradictory, although similar results have been reported by Tsou [33]. The decreased percentage of Mac-3-expressing cells indicated that differentiation of macrophage precursors was inhibited after *P. yoelii* infection and that *P. yoelii* infection could inhibit murine leukaemia WEHI-3 cell proliferation. The increase in macrophage activity may have been induced by the parasite, as Plasmodium infection has been shown to significantly enhance the phagocytic activity of macrophages due to increased IFN-γ expression [34, 35], and in the present study, IFN-γ secretion was found to be increased in mice after *P. yoelii* infection.

## Conclusions

Malaria parasite infection can enhance immune responses, including innate and adaptive anti-leukaemia responses, thereby significantly decreasing the number of myeloblasts and inhibiting neoplasm proliferation in a mouse model. The results suggest that the malaria parasite may represent a novel strategy or therapeutic vaccine vector for an immune-based therapy for leukaemia.

Although malaria parasite infection elicits an anti-leukaemia effect, the use of parasitic infections against leukaemia under clinical conditions is impossible. However, cancers and parasites may share common antigens, which may explain why certain parasites exhibit anticancer activity [10]. Therefore, the active antigens of malaria parasites for leukaemia immunotherapy are worthy of further study and identification.

## Additional files

Additional file 1: Multilingual abstracts in the five official working languages of the United Nations. (PDF 492 kb)

## Abbreviations

CD: Cluster of differentiation
ELISA: Enzyme-linked immunosorbent assay
FBS: Fetal bovine serum
FITC: Fluorescein isothiocyanate
H&E: Hematoxylin and eosin
IFN-γ: Interferon-gamma
NK: Natural killer cells
OD: Optical density
PI: Propidium iodide
TNF-α: Tumor necrosis factor-alpha
