# Supplementary material for: Plasmodium yoelii infection inhibits murine leukaemia WEHI-3 cell proliferation in vivo by promoting immune responses
Source: Infect Dis Poverty. 2018 May 16;7:48. doi: 10.1186/s40249-018-0433-4 (PMC5954458; doi:10.1186/s40249-018-0433-4)
Supplement: Supplementary file 1 — Multilingual abstracts in the five official working languages of the United Nations. (PDF 492 kb) [file 40249_2018_433_MOESM1_ESM.pdf]

Translation of the abstract into the five official working languages of the United Nations

*يتسببُ طفيل البلازمود المنجلي*بالعدوىالتي تُثبطُ خلية سرطان الدم (WEHI-3) بالفئران إذ تنتشر بالكائنات الحيةعبر تعزيز استجابات الجهاز المناعي

قدمه: تشن تشن تونغ, وتشنغ مينغ فانغ، وتشى تشانغ، ويون زان، ويو تشانغ، ووان فانغ جيانغ، وشياو هو، ويونغ لونغ لي وتينغ وانج

الملخص

المعلومات الأساسية:سرطان الدم هو اضطراب خبيث يُصيب كريات الدم البيضاءواحتمالات الوفاة جرّاء هذا المرض عالية،و أساليب العلاج الحالية لهذا المرض غير مُرضية. لذلك،يجب أن تُستحدث استراتيجيات علاجية جديدة لسرطان الدم. فقد أثبتت العدوى الناجمة عن طفيلي الملاريا فعاليتها في محاربة بعض الأورام الخبيثة بالتجارب المختبرية على الحيوانات.

الطرق: لغرض بيان النشاط المضاد لسرطان الدم لدى طفيلي الملاريا *طفيل البلازمود المنجلي*(عدوى البلازمود المنجلي)، قمنا بتحليل نسبة الخلايا بالمستقبلات المُتممة CD3, وCD19, وCD11b,و Mac-3 عبر قياس التدفق الخلوي، وقياس مستويات بروتينات الإنترفيرون غاما (IFN-γ) وعامل نخر الورم ألفا (TNF-α) الموجودة في عينات المصل الفردية عبر تقنية المقايسة الامتصاصية المناعية للإنزيم المرتبط، وقياس النشاط البلعمي بالخلايا البلعمية الكبيرة والنشاط بالخلية الفاتكة الطبيعية عبر قياس التدفق الخلوي.

النتائج: لقد وجدنا أن*العدوى الناجمة عن طفيل البلازمود المنجلي تُحدُ*بقدرٍ كبير من نمو خلايا (WEHI-3)في الفئران. وبالإضافة إلى ذلك، انخفض معدل تغلغل الخلايا السرطانية في الكبد والطحال لدى فئران التجربة بدرجة كبيرة. كما أننا بيَّنا الكيفية التي يمكن بها لعدوى طفيلي الملاريا إثارة النشاط المضاد لسرطان الدم عبر تعزيز استجابات الجهاز المناعي، ويشمل ذلك زيادة مُعلمات أسطح الخلايا التائية (CD3) والخلايا البائية (CD19)؛ وخفض مُعلمات أسطح الخلايا الوحيدة (CD11b) والخلايا البلعمية الكبيرة (Mac-3)؛ وتحفيز إفراز بروتينات الإنترفيرون غاما (IFN-γ) وعامل نخر الورم ألفا (TNF-α)؛ وزيادة نشاط الخلية الفاتكة الطبيعية والخلايا البلعمية الكبيرة

الاستنتاجات: تعملُ العدوى الناجمة عن طفيلي الملاريا على خفض عدد خلايا الجذعة النخاعية بقدرٍ كبير وتُثبطُ انتشار الأورام في الفئران. إضافةً إلى ذلك، تُثبطُ عدوى طفيلي الملاريا سرطان الدم لدى الفئران عبر تعزيز استجابات الجهاز المناعي.

Translated from English version into Arabic by Bashaier Allam and Saher Salama, through

TRANSLATORS WITHOUT BORDERS

约氏疟原虫感染促进免疫反应来抑制小鼠白血病体内 WEHI-3 细胞增殖

Zhen-Zhen Tong, Zheng-Ming Fang, Qi Zhang, Yun Zhan, Yue Zhang, Wan-Fang Jiang, Xiao Hou, Yong-Long Li and Ting Wang

引言：白血病是一种死亡率很高的恶性血液疾病，虽然治疗方法不断改善，但疗效并不尽如人意。因此，现急需发展新的治疗方法。而现已在动物实验中证实，疟原虫感染能有效抑制某些肿瘤。

方法：为了研究疟原虫的抗白血病活性，我们用流式细胞仪检测每组小鼠外周血中的 T 细胞表面标志 CD3、B 细胞表面标志 CD19、单核细胞表面标志 CD11b 和巨噬细胞表面标志 Mac-3 的比例；用 ELISA 试剂盒检测血清中 IFN-γ 和 TNF-α 的含量；用流式细胞仪检测腹

腔巨噬细胞的吞噬活性和 NK 细胞的杀伤活性。
结果：疟原虫感染明显抑制了小鼠体内白血病细胞 WEHI-3 的增殖，并使白血病小鼠肝和脾中浸润的肿瘤细胞明显减少。疟原虫感染可提高白血病小鼠 CD3 和 CD19 的水平，降低 CD11b 和 Mac-3 的水平；诱导 IFN-γ 和 TNF-α 的分泌；增强巨噬细胞和 NK 细胞的活性。以上结果表明，疟原虫感染可通过促进免疫反应来发挥抗白血病效应。
结论：疟原虫感染可显著抑制白血病小鼠中肿瘤细胞的分化和增殖。且疟原虫感染通过促进免疫反应来发挥抗白血病效应。

Translated from English version into Chinese by Zhen-Zheng Tong and Ting Wang

L'infestation par *Plasmodium yoelii* inhibe la prolifération des cellules leucémiques WEHI-3 murines *in vivo* en promouvant les réponses immunitaires

Zhen-Zhen Tong, Zheng-Ming Fang, Qi Zhang, Yun Zhan, Yue Zhang, Wan-Fang Jiang, Xiao Hou, Yong-Long Li and Ting Wang

Résumé
Contexte : La leucémie est une pathologie maligne des leucocytes, dont le taux de mortalité est élevé et contre laquelle les traitements actuels donnent des résultats peu satisfaisants. De nouvelles stratégies thérapeutiques doivent donc être mises au point contre la leucémie.Or il s'est avéré que l'infestation par le parasite agent du paludisme donnait de bons résultats pour combattre certaines tumeurs dans des expérimentations sur l'animal.
Méthodes : Afin de démontrer l'activité antileucémique du parasite *Plasmodium yoelii* (*P. yoelii*), agent du paludisme, la proportion de cellules CD3, CD19, CB11b et Mac-3 a été analysée par cytométrie de flux, les taux d'IFN-γ et de TNF-α dans les échantillons de sérum individuels ont été mesurés par immunosorption enzymatique et l'activité phagocytaire des macrophages et des lymphocytes NK a été mesurée par cytométrie de flux.
Résultats : Nous avons constaté que l'infestation par *P. yoelii* atténuait significativement la prolifération des cellules WEHI-3 chez les souris. En outre, l'infiltration du foie et de la rate par les cellules tumorales était nettement réduite. Nous avons également démontré que l'infestation par le parasite palustre suscitait une activité antileucémique en promouvant les réponses immunitaires : augmentation des marqueurs de surface des lymphocytes T (CD3) et B (CD19), diminution des marqueurs de surface des monocytes (CD11b) et des macrophages (Mac-3), induction de la sécrétion d'IFN-γ et de TNF-α et augmentation de l'activité des lymphocytes NK et des macrophages.
Conclusions : L'infestation par le parasite agent du paludisme diminue significativement le nombre de myéloblastes et inhibe la prolifération tumorale chez les souris. En outre, elle freine la leucémie murine en promouvant les réponses immunitaires.

Translated from English version into French by Suzanne Assenat and Sophie N, through

Инфицирование *Plasmodium yoelii* подавляет размножение клеток WEHI-3 мышиного лейкоза *в живом организме* за счёт стимуляции иммунных реакций

Чжэнь-Чжэнь Тонг, Чжэн-Минь Фан, Ци Чжан, Юнь Чжан, Юэ Чжан, Ван-Фан Цзян, Сяо Хоу, Юн-Лонг Ли и Тинг Ванг

Аннотация
Исходные данные: лейкоз — это злокачественная патология лейкоцитов с высоким показателем смертности, причём существующие методы лечения этого заболевания не приносят ожидаемых результатов. Поэтому необходимо разработать новые терапевтические стратегии лечения лейкоза. В ходе экспериментов с животными выяснилось, что инфицирование малярийным плазмодием является эффективным способом борьбы с определенными видами новообразований.
Методы: для демонстрации антилейкозного действия малярийного плазмодия *Plasmodium yoelii* (*P. yoelii*) было проанализировано соотношение клеток CD3, CD19, CD11b и Mac-3 методом проточной цитометрии, были измерены уровни интерферон-гамма (IFN-γ) и фактора некроза опухолей-альфа (TNF- α) в отдельных пробах сыворотки методом ферментного иммуносорбентного анализа, а также фагоцитарная активность макрофагов и активность природных клеток-киллеров методом проточной цитометрии.
Результаты: мы выяснили, что инфицирование плазмодием *P. Yoelii* значительно ослабляет рост клеточной линии WEHI-3 у мышей. Кроме того, заметно сократилось проникновение опухолевых клеток в печень и селезёнку мышей. Мы также доказали, что инфицирование малярийным плазмодием вызывает антилейкозное действие за счёт стимуляции иммунных реакций, включая увеличение поверхностных маркеров Т-клеток (CD3) и В-клеток (CD19); уменьшение поверхностных маркеров моноцитов (CD11b) и макрофагов (Mac-3); стимуляцию секреции IFN-γ и TNF-α; и повышение активности природных клеток-киллеров и макрофагов.
Заключения: заражение малярийным плазмодием значительно снижает количество миелобластов и препятствует разрастанию новообразований у мышей. Помимо этого, инфицирование малярийным плазмодием сдерживает развитие мышиного лейкоза за счёт стимуляции иммунных реакций.

Translated from English version into Russian by Kate Kazyk and Galina Dmitrieva, through

La infección por Plasmodium yoelii inhibe la proliferación de células WEHI-3 de la leucemia murena_in vivo_ al favorecer la respuesta inmunitaria

Zhen Zhen Tong Fang Zheng Ming, Qi Zhang, Yun Zhan, Yue Zhang, Jiang Wan Fang, Xiao Hou, Li Yong-largo y Ting Wang

Resumen

Antecedentes: La leucemia es un trastorno maligno de los leucocitos con una elevada tasa de letalidadylos tratamientos actuales para esta enfermedad son insatisfactorios. Por lo tanto,se deben desarrollar nuevas estrategias terapéuticas para la leucemia. Se ha demostrado, en experimentos con animales, que la infección causada por el parasito del paludismo es eficaz para combatir ciertos neoplasmas.

Métodos:para demostrar la actividad antileucémica de la infección causada por el parásito del paludismo*el Plasmodium yoelii* (*P. yoelii*), se analizaron las proporciones de células CD3, CD19, CD11b y Mac-3 por citometría de flujo; se midieron los niveles de IFN-γ y TNF-α de muestras séricas individuales mediante la técnica de inmunoabsorción enzimática y, la actividad fagocítica de los macrófagos junto con las células (NK) se analizaron por citometría de flujo.

Resultados: encontramos que *la infección por P. yoelii* atenuó de forma significativa el crecimiento de células WEHI-3 en ratones. Además, se redujo notablemente la infiltración de células tumorales en el hígado murino. También mostramos que la infección causada por el parásito del paludismo desencadenó una actividad antileucémica al favorecer respuestas inmunitarias, tales como: un aumento de los marcadores de superficie de células T (CD3) y células B (CD19);una disminución de los marcadores de monolitos (CD11b) y macrófagos (Mac-3); una inducción de la secreción de IFN-γ y TNF-α y un aumento de células NK y dela actividad macrófaga.

Conclusión:la infección causada por el parásito del paludismo disminuye de manera significativa el número de mieloblastos e inhibe la proliferación de neoplasma en los ratones. Además, esta infección inhibe la leucemia murina al favorecer la respuesta inmunitaria.

Translated from English version into Spanish by FTP zqa Lissy Sugey, through

TRANSLATORS
WITHOUT BORDERS
